# Species and age related differences in the type and distribution of influenza virus receptors in different tissues of chickens, ducks and turkeys

**DOI:** 10.1186/1743-422X-7-5

**Published:** 2010-01-12

**Authors:** Smitha PS Pillai, Chang W Lee

**Affiliations:** 1 Food Animal Health Research Program, Ohio Agricultural Research and Development Center, The Ohio State University, Wooster, Ohio 44691, USA; 2 Department of Veterinary Preventive Medicine, College of Veterinary Medicine, The Ohio State University, Columbus, Ohio 43210, USA

## Abstract

We undertook one of the most detailed studies on the distribution of α2,3 sialic acid (SA)-galactose (gal) (avian type) and α2,6SA-gal (human type) receptors on different tissues of chickens, ducks and turkeys of varying age groups. On the tracheal epithelium, all 3 bird species expressed strong positive staining (80-90%) for α2,3SA-gal receptors in the 3 different age groups. In addition, a lesser amount of α2,6SA-gal receptors (30-90%) were observed with slight differences in distribution with age and species. The epithelium of the small and large intestine of turkeys and ducks showed negligible staining for α2,6SA-gal receptors whereas the large intestine consistently showed 40-70% positive staining for α2,3SA-gal receptors. In contrast, a greater amount of staining for α2,3SA-gal (50-80%) and α2,6SA-gal (20-50%) receptors were observed along the epithelium of small and large intestine of chickens. Kidney and esophagus sections from the 3 bird species also expressed both avian and human type receptors. In other tissues examined, brain, breast muscles, bursa, spleen, cecal tonsils and oviduct, human type receptors were absent. Though different viral and receptor components may play roles in successful viral replication and transmission, understanding the receptor types and distribution in different tissues of domestic birds might be good initial tool to understand host factors that promote successful influenza viral infection.

## Introduction

Wild aquatic birds are considered to be the natural reservoir of influenza viruses. They have been implicated as the source of influenza viruses for all other species of birds and mammals [1,2]. In wild aquatic birds, influenza viruses are believed to have tropism for the digestive tract and follow a fecal oral mode of transmission [3]. Influenza viruses in wild aquatic birds are believed to possess a strict binding preference for sialic acids (SA) linked to galactose (Gal) through α2,3 linkages [4]. Previous immunohistochemical studies using plant lectins revealed the presence of α2,3SA-gal residues and no detectable expression of α2,6SA-gal receptors in duck intestinal cells [5,6]. Similarly, human viruses were found not to bind to plasma membranes isolated from duck intestinal cells thereby confirming the absence of α2,6SA-gal linked sialyloligosaccharides on duck intestinal epithelial cells [5]. Though not natural hosts, many land based poultry like chickens, turkeys and quail have been found to support the replication and transmission of a variety of influenza subtypes [7]. Recent studies as well as the human infections caused by H5N1 and H9N2 viruses suggested that domestic poultry can be immediate precursors as well as potential intermediate hosts, like pigs, for influenza viruses. α2,3SA-gal and α2,6SA-gal linked receptors have been detected in the tracheal epithelium of chickens and quail suggesting that they can be infected with avian and mammalian viruses and serve as adaptation hosts for changing the receptor preference of avian viruses from α2,3SA-gal to α2,6SA-gal [8]. Though turkeys are frequently infected with avian and swine influenza viruses, reports on the receptor profile of tissues from turkeys are lacking. Similarly, few studies have been undertaken to understand the distribution and type of receptors from different tissues of domestic chickens and ducks. Influenza viruses in domestic birds are found to evolve faster than aquatic bird viruses and are characterized by the presence of additional carbohydrates on hemagglutinin and deletions in the stalk of neuraminidases. These findings may have implications for the receptor binding and sialidase activity of the virus and suggest that the spectrum of sialic acid containing receptors on different bird species is not identical [5].

Studies on the type and distribution of receptors in different tissues of domestic poultry are still incomplete. In this study, we examined the presence and type of α2,3SA-gal and α2,6SA-gal receptors on different tissues of domestic poultry that included chickens, ducks and turkeys. We also looked at the age related differences in the distribution of receptors in these 3 bird species.

## Materials and methods

### Birds and tissues analyzed

White Leghorn chickens (Charles River Laboratories, Inc. Wilmington, MA), commercial Pekin ducks (Ridgeway Hatcheries, Inc. LaRue, Ohio) and Eggline turkeys (maintained at Ohio Agricultural Research and Development Center, Wooster, Ohio) of 3 different age groups (1-day-old, 2-4-week-old and 52-60-week-old adult layer birds) were used in the present study. Throughout the study, the birds were handled according to an approved Institutional Animal Care and Use Committee guideline. We collected different tissues that included trachea, lung, spleen, bursa, cecal tonsil, esophagus, portions of small and large intestines, and kidney from the 3 species of birds.

### Immunohistochemistry for the detection of receptors using plant lectins

We examined different tissues of poultry for the presence of receptors by employing two specific lectins, Maackia amurensis agglutinin (MAA) for α2,3SA-gal receptors and Sambucus nigra agglutinin (SNA) for α2,6SA-gal receptors (DIG Glycan Differentiation Kit, Roche Applied Science, Mannheim, Germany). Paraffin embedded tissue sections were deparaffinized and immersed in 3% hydrogen peroxide to eliminate the endogenous peroxidase activity. The sections were treated with blocking agent to avoid nonspecific staining and then incubated with digoxigenin (DIG)-labelled MAA or SNA (1 μg/μl) at 4°C overnight. Slides prepared with serial sections of the same tissue were incubated with PBS instead of lectin as negative controls. After two washes in phosphate-buffered saline (PBS), the sections were incubated with peroxidase-labelled anti-DIG FAb fragments (Roche Applied Science) for 1.5 h at 37°C. Lectin binding was visualized using DAB (3, 3' -diaminobenzidine-tetrahydrochloride) substrate (Roche diagnostics GmbH, Mannheim, Germany) and slides were counterstained with hematoxylin.

## Results

The receptor distribution in different tissues was determined as the average percentage of positive staining observed by visual examination of 3 different fields of the tissue from at least 3 birds of each species of specific age as observed under 200× magnification of light microscope. The staining intensity, that correspond to the number of sialic acid moieties stained per cell, was relatively compared and assigned as mild (+), moderate (++), strong (+++) or very strong (++++).

### Differences in receptor distribution in the respiratory tracts of chickens, ducks and turkeys with age

**Figure 1:**
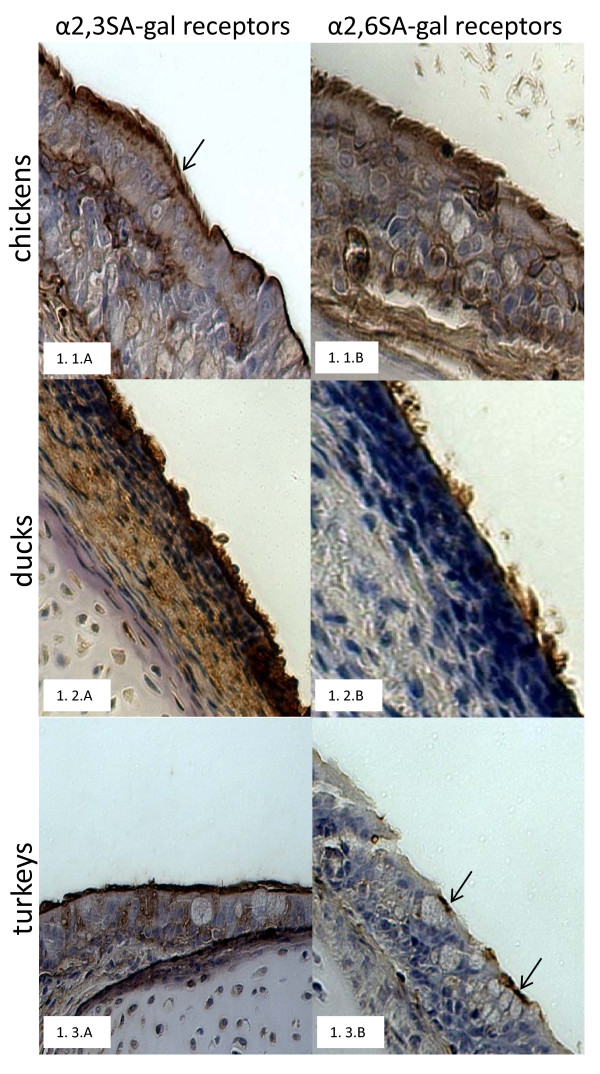
**Distribution of α2,3SA-gal and α2,6SA-gal receptors along the tracheal epithelium of 4-week-old chickens (1.1.A, 1.2.B), 2-week-old ducks (1.2.A, 1.2.B) and 3-week-old turkeys (1.3.A, 1.3.B) using plant lectins, MAA and SNA, respectively**.

In all 3 bird species, the tracheal epithelium showed the predominance of α2,3SA-gal receptors. Strong positive staining (80-90%, ++++) for α2,3SA-gal receptors was visible throughout the tracheal epithelial lining in the 3 bird species (Fig. 1). In day-old ducks and chickens, 90% (+++), and 60% (++) of the lining cells, respectively, were positive for the α2,6SA-gal receptors. In contrast, in day-old turkeys, approximately, 20% of the tracheal epithelial cells showed moderate positive staining (++) for α2,6SA-gal receptors (Table 1).

**Table 1:** The distribution and intensity of a2,6SA-gal (stained by MAA) and a2,3SA-gal (stained by SNA) receptors on different tissues of 1-day-old, 2-4-week old and adult layer chickens, ducks and turkeys

		**Species**																	
	
**Tissues**		**1-day**		**2-4-weeks**		**layers**		**1-day**		**2-4-weeks**		**layers**		**1-day**		**2-4-weeks**		**layers**	
									
		MAA % ^a^ Int^b^	SNA % Int	MAA % Int	SNA % Int	MAA % Int	SNA % Int	MAA % Int	SNA % Int	MAA % Int	SNA % Int	MAA % Int	SNA % Int	MAA % Int	SNA % Int	MAA % Int	SNA % Int	MAA % Int	SNA % Int
Trachea		90 ++++	60 ++	90 +++	60 +++	80 +++	80 ++	90 ++++	90 ++++	90 ++++	90 +++	90 ++++	90 ++++	90 ++++	20 +++	90 ++++	70 +++	90 ++++	30 +++
Bronchi		90 ++++	60 ++	80 +++	60 +++	60 ++	-^c^	90 ++++	90 ++++	90 ++++	90 ++++	90 ++++	90 ++++	90 +++	50 ++	90 ++++	60 ++	50 +	10 +
Small intestine		60 ++	10 ++	40-60 ++	20 ++	30 ++	-	-	-	-	-	25 +++	-	-	-	10 +++	-	-	-
Large intestine		80 +++	20 ++	70 +++	30-50 +++	80 +++	10 ++	>50 +++	5-10 +	35 +++	-	40-50 +++	-	70 +++	-	40 +++	-	50 +++	-
Kidney		70 ++++	20 ++	60 +++	30 ++	60 ++	50 ++	40 ++++	20 ++	60 ++++	10 ++	60 ++++	30 ++	60 ++++	-	50 ++++	20 ++	50 +++	20 ++
Oviduct		na^d^	na	na	na	80 +++	-	na	na	na	na	90 ++++	-	na	na	na	na	90 ++++	-

^a^: The receptor distribution in different tissues determined as the average percent of positive staining observed by visual examination of 3 different fields of the tissue from at least 3 birds of each species of specific age as observed under 200× magnification using a light microscope

^b^: Intensity of staining observed, that corresponds approximately to the number of sialic acid molecules stained per cell, expressed as +(mild), ++(moderate), +++(strong) and ++++(very strong)

^c^: No staining observed

^d^: Not applicable

In day-old ducks and chickens, similar results as for trachea were observed for bronchial epithelial cells, with 90% of the epithelial cells staining positive (++++) for α2,3SA-gal receptors and lesser intensity (+++) and fewer percent (60-90%) of cells showing positive staining for α2,6SA-gal receptors. Minor difference was observed in turkey poults with lower percentage (50%) of α2,6SA-gal receptors on the bronchial epithelium with a lower staining intensity (++).

The respiratory epithelium of 2-4 week old chickens and ducks gave similar results as in 1-day-old birds. However in 2-4 week old turkeys there was an increase of approximately 50% of cells staining positive for the human type receptors in tracheal epithelium in comparison to the sections from day-old turkey poults.

The α2,3 and α2,6SA-gal receptor distribution in the trachea, bronchi and lungs of layer ducks was similar to the distribution in 1-day-old as well as 2-4-week-old ducks. In chickens, an increase (from 60% to 80% positive cells); and in turkeys, a decrease (from 70% to 30% positive cells) in staining for α2,6SA-gal receptors was observed along the tracheal epithelium. The bronchial epithelium of layer chickens did not show the presence of human type receptors. With the exception of bronchial epithelium, sections prepared from different parts of the lung were negative for the presence of both α2,3SA-gal or α2,6SA-gal receptors in different age groups of the 3 bird species.

### Differences in receptor distribution along the epithelium of small and large intestine of chickens, ducks and turkeys with age

In day old ducks, less than 5% (+++) of the epithelial cells of small intestine showed positive staining for the avian type receptors with no detectable presence of human type receptors while no staining for both receptors was observed in turkey poults. In contrast, in day-old chickens, approximately 60% (++) positive staining was observed for α2,3SA-gal receptors, with less positive staining (10%, ++) for α2,6SA-gal receptors.

The epithelial cells of large intestine showed the presence of avian type receptors in day-old birds of all 3 species, with chickens also showing the presence of mammalian receptors (20%, ++). The distribution of the avian receptors varied from 40-70% in most of the epithelial cells of large intestine in the 3 bird species (Table 1).

We did not observe the presence of either type of receptors in the epithelium of small intestine of 2-week-old ducks. However with 3-week-old turkeys, epithelial cells from jejunum and ileum showed positive staining for avian type receptors (10%, +++). In 3-week-old chickens, epithelial cells of jejunum (40%, ++) and ileum (60%, +++) showed higher percentage of positive staining for α2,3SA-gal receptors. The epithelial cells from ileum of chickens also showed presence of α2,6SA-gal receptors (20%, ++).

**Figure 2:**
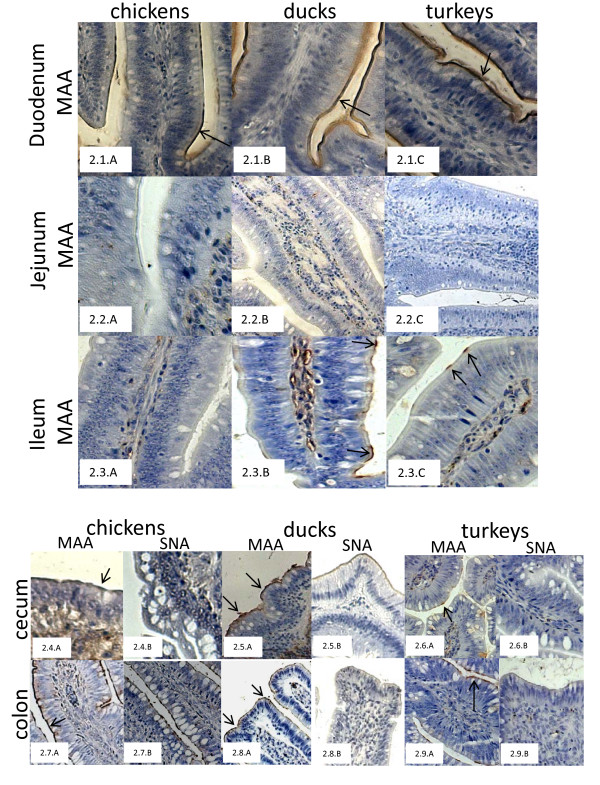
**Distribution of α2,3SA-gal and α2,6SA-gal receptors along the jejunum of 4-week-old chickens (2.1.A-C), 2-week-old ducks (2.2.A-C) and 3-week-old turkeys (2.3.A-C) using plant lectins, MAA and SNA, respectively**. Sections of ceca from 4-week-old chickens (2.4.A, B), 2-week-old ducks (2.5.A, B) and 3-week-old turkeys (2.6.A, B) stained with MAA and SNA respectively. Sections of colon from 4-week-old chickens (2.7.A, B), 2-week-old ducks (2.8.A, B) and 3-week-old turkeys (2.9.A, B) stained with MAA and SNA, respectively.

The epithelial cells of large intestine showed 30-50% staining (+++) for the presence of α2,3SA-gal receptors in 2-week-old ducks and turkeys with no positive staining for human type receptors. In 4-week-old chickens, along the epithelium of large intestine, a higher percentage of positive staining (70%, +++) was observed for avian type receptors along with the presence of human type receptors (30-50%, +++) (Fig. 2).

The epithelial cells of small intestine of layer chickens and ducks showed positive staining for avian receptors (25-30%, +++), however, sections of small intestine from breeder turkeys were negative for the presence of avian type receptors. Layer chickens showed higher percentage of positive staining for avian type receptors along the epithelium of large intestine (80%, +++) in comparison to ducks (40-50%, ++ to +++) or turkeys (50%, +++). No human type receptors were observed in small or large intestine.

### Differences in distribution of receptors in other tissues examined

In day-old birds, the tubular cells of the kidney showed positive staining for α2,3SA-gal and α2,6SA-gal receptors in the 3 bird species. Approximately, 40-70% of the cells showed very strong positive staining (++++) for the presence of avian type receptors. Less than 30% of the cells were positive for α2,6SA-gal receptors and the staining intensity was moderate (++).

**Figure 3:**
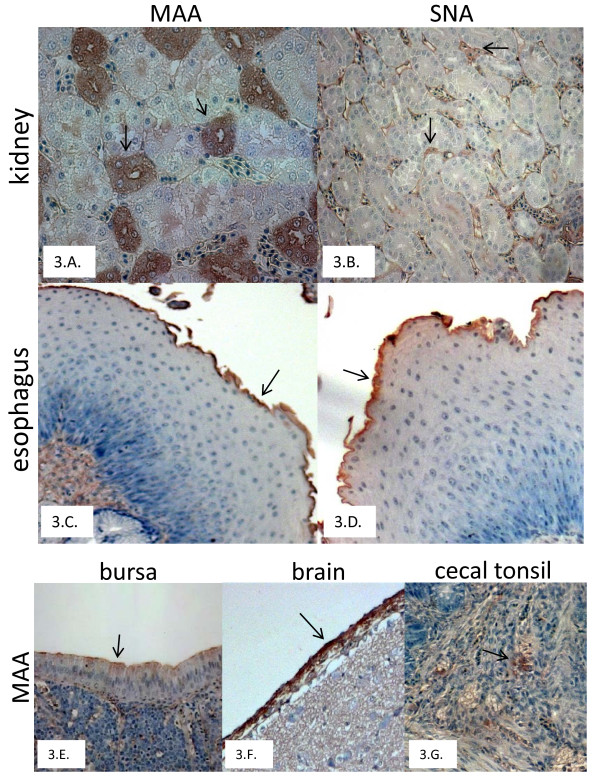
**Sections of kidney (3.A, B) and esophagus (3.C, D) from 4-week-old chickens stained with MAA and SNA, respectively**. Sections of bursa (3.E), brain (3.F), cecal tonsil (3.G) from 4-week-old chickens stained with MAA.

Similar to the 1-day-old birds, the 2-4-week-old birds and layer birds of the 3 species showed strong staining (++++) in the tubular cells of the kidney (50-60%) for the avian type receptors. The tubular cells also showed positive staining for the human type receptors, although the strained cells was less (10-30%) and mild to moderate intensity (+ to ++) of staining was observed (Fig. 3A and 3B).

Among the layer birds of the 3 species tested, all the sections of the oviduct including the infundibulum, magnum, isthmus and the uterus showed high intensity of positive staining (80-90%, ++++) for the avian type receptors. These sections did not give any positive staining for the human type receptors. Results of receptor staining for turkey oviduct sections were previously reported [9].

No human type receptors were detected in other organ sections (brain, breast muscles, bursa, spleen, and cecal tonsils) tested. In the brain, positive staining for avian type receptors was found in the meningeal layer surrounding the brain (Fig. 3F). The sections of the esophagus gave strong positive staining for both avian and human type receptors along the mucosal epithelium (Fig. 3C and 3D). Though influenza viral replication has been demonstrated in muscles and lymphoid tissues (bursa, thymus and spleen) by immunohistochemistry, avian or human type receptors were not detected in these tissue sections.

## Discussion

Influenza viruses attach to host cells through interactions of the viral hemagglutinin with sialic acid terminated oligosaccharide residues on host cells. These interactions determine to a large extent the host range and successful interspecies transmission of influenza viruses [10]. Sialic acids, a family of 9-carbon acid sugars were identified and are still believed to be major receptor determinants of influenza viruses [11]. Using specific sialic acid determinants generated by sialyltransferases, human and avian viruses were found to preferentially bind to α2,6SA-gal (human type) and α2,3SA-gal (avian type) receptors, respectively [12,13].

The presence of avian and human type receptors on the tracheal epithelium of the 3 species of birds even at one day of age, indicate that both avian and human influenza viruses may utilize these receptors for binding to initiate infections. The presence of avian receptors in the trachea and bronchial epithelium and their absence in other parts of lung support previous findings that influenza viruses mainly localize in the upper respiratory tracts in domestic birds [1]. Chicken tracheal epithelial cells have been previously shown to posses both types of receptors and chickens have been proposed to be potential intermediate hosts in the interspecies transmission of influenza viruses [14].

Equal intensity of strong positive staining for avian and human type receptors observed in the trachea of ducks of the 3 age groups was an interesting finding, especially considering the dominant presence of α2,3SA-gal receptors in epithelial cell of the large intestine. The presence of avian type receptors on the tracheal epithelium of ducks is supported by their susceptibility to low and highly pathogenic influenza viruses and successful oropharyngeal shedding [15]. Also surveillance studies report high rates of viral recovery from tracheal swabs similar to cloacal swabs from ducks [1,16,19]. A recent study employing immunoflourescence staining also indicated the presence of α2,3SA-gal and α2,6SA-gal receptors on duck tracheal epithelial cells [20].

With turkeys, studies on the receptor distribution profile from the tracheal epithelium are lacking. Turkeys have been found to be naturally and experimentally infected with influenza viruses of avian and mammalian origins [16,21,25]. The presence of avian and human type receptors in turkeys along with their higher susceptibility to wild and domestic bird origin and swine viruses strengthens the argument that turkeys, like chickens and quail can be potential intermediate hosts for interspecies transmission and spread of reassortant viruses between birds and humans.

Differences in percent staining of avian and human type receptors were seen along the tracheal epithelia in different age groups of chickens, ducks and turkeys. However, it is not clear if such percentages have an effect on the infection with viruses from different sources or if a minimum percent of receptors is enough to initiate infections.

The distribution and intensity of receptors in the bronchial epithelium of the 3 bird species was similar to the results observed for tracheal epithelium. Failure to detect receptors in different parts of the lung tissues does not indicate absence of influenza virus replication in lung tissues of domestic birds. Many high and low pathogenic influenza virus infections of domestic and live bird market poultry have been found to infect lungs and viral antigen has been demonstrated in lungs tissues [26,27]. The presence of lung infection in conjunction with failure to detect receptors might indicate that the distribution of receptors in the respiratory tract might not be as clear cut as we observe using lectin histochemistry and that other host and viral components might play a role [28,29].

With the intestinal sections, only chicken intestinal epithelial cells exhibited avian and human type receptors among the 3 bird species tested. With turkeys and ducks, only avian type receptors were predominant and were mostly restricted to the large intestine. Few previous reports indicate high frequency of viral isolation from cloaca, jejunum and ileum following experimental inoculation of wild waterfowl origin viruses in chickens [17,30]. Our results are in agreement with previous studies that reported the presence of α2,3- and α2,6SA-gal receptors on chicken colon [31] and absence of SNA staining in duck intestinal cells [6]. Also, chicken duodenum was not found to express α2,6SA-gal receptors as previously reported [27]. Studies by Wan and Perez [8] reported large amounts of positive cells for α2,3SA-gal residues along the chicken duodenal sections, especially in crypts. Our study revealed positive staining for α2,3SA-gal receptors along the jejunum and ileum and α2,6SA-gal receptors in ileal sections of chicken intestines, with no positive staining for either type of receptors along the duodenal sections of chickens. We do not know if such discrepancies in results were due to the different MAA isoforms that were employed in these studies. The use of different breeds of birds within the same species as well as differences in tissue processing techniques may also account for the different staining results observed.

Kidney sections from the 3 bird species were found to be positive for the presence of avian and human type receptors. Many influenza viruses have been found to be nephrotropic following infection [9,32,33]. Madin Darby canine kidney (MDCK) cell line and primary chicken embryonic kidney cells have been found to support efficient replication of influenza viruses [34]. Our results indicate that kidney cell lines from domestic poultry of the 3 age groups that we studied could be used for influenza viral propagation. This may offer the additional advantage of species specificity with the avian cell lines and use of adult birds in place of chicken embryos alone for viral propagation. In addition to kidney, we observed the presence of both avian and human type receptors along the esophageal mucosa indicating that influenza viruses can attach and possibly replicate in the upper digestive tract which is an important portal of viral entry and supports the fecal-oral transmission route of influenza viruses.

The oviduct from all species of birds showed the presence of α2,3SA-gal linked receptors. Influenza infections have been associated with lowered egg production in layer chickens and breeder turkeys [9,17]. It is possible that the viruses utilize the α2,3 linked sialic acid receptors in the oviduct for binding and subsequent infections. Our previous studies in breeder turkeys using a triple reassortant turkey virus, A/turkey/Ohio/04 (H3N2), showed that the virus preferentially replicates in the oviduct of breeder turkeys in comparison to the respiratory or digestive tracts and result in drastic declines in egg production in breeder turkeys [9]. This study also showed an exact match between the presence of α2,3SA-gal receptors and viral antigen in duplicate sections of the oviduct indicating that the viruses might utilize these receptors for virus-cell interactions.

The absence of receptors in tissues like spleen, brain, cecal tonsils analyzed in this study does not necessarily indicate absence of infection with influenza viruses especially following infection with highly pathogenic isolates indicating again that receptor distribution might not be as clear cut as observed with lectin immunonochemistry. Highly pathogenic avian influenza isolates have been found to consistently localize to brain and pancreas of infected birds [24,35,36]. Viral antigen has also been demonstrated from muscle tissues of experimentally infected ducks [37]. A high frequency of viral recovery has been demonstrated from the bursa of chickens following experimental inoculation using waterfowl origin influenza viruses [27]. Experimental inoculation of highly pathogenic viruses into chickens has revealed histological lesions consisting of necrosis and inflammation in cloacal bursa, thymus, spleen, heart muscle, brain along with lesions in pancreas and lung tissues [27]. Highly pathogenic avian influenza viruses have also been isolated from duck meat following infection [19]. Even in the demonstrable absence of receptors, documentation of viral replication in these organs indicate that yet to be known receptor determinants might be involved. These findings also indicate the shortcomings of receptor studies using lectin histochemistry. Nevertheless, the presence of α2,3SA-gal and α2,6SA-gal receptors along their tracheal epithelium, bronchus, esophagus, and intestinal tract might indicate the possibility of adaptation of wild bird viruses in domestic turkeys, ducks and chickens and occasional emergence of viruses with different receptor preference and an enhanced propensity for transmission to different species.

## Competing interests

The authors declare that they have no competing interests.

## Authors' contributions

SPSP participated in the design of the study, performed the study, read the immunohistochemistry slides, and drafted the manuscript. CWL conceived of the study, participated in its design and coordination, and completed the manuscript. All authors read and approved the final manuscript.
